# Supplementary material for: Heterogeneity of Modern Contraceptive Use among Urban Slum and Nonslum Women in Kinshasa, DR Congo: Cross-Sectional Analysis
Source: Int J Environ Res Public Health. 2021 Sep 6;18(17):9400. doi: 10.3390/ijerph18179400 (PMC8430884; doi:10.3390/ijerph18179400)
Supplement: Supplementary file 1 [file ijerph-18-09400-s001.zip › ijerph-1350019-supplementary.pdf]

International Journal of
Environmental Research and Public Health

MDPI

Supplementary Materials

Table S1. Socio-demographic characteristics of women in Kinshasa by residence, stratified by age group, 2020.

	Slum neighborhood				Nonslum Neighborhood			
	N	15-19 (n=444)	20-24 (n=410)	25-49 (n=1078)	N	15-19 (n=130)	20-24 (n=129)	25-49 (n=373)
		%	%	%		%	%	%
**Education**								
None/primary education	175	5.2	4.3	12.4	33	4.9	3.7	5.9
Secondary education	1449	89.8	72.2	70.0	433	80.7	59.8	67.2
Tertiary education	308	4.9	23.5	17.6	166	14.4***	36.5***	26.9**
Total	1932	100.0	100.0	100.0	632	100.0	100.0	100.0
**Marital status**								
Never married	924	94.7	74.5	18.4	349	97.5	83.2	30.7
Currently married	876	4.3	24.0*	70.4***	236	2.5	15.1	57.3
Divorced/widowed	131	1.0	1.4	11.2	47	0.0	1.8	12.0
Total	1932	100.0	100.0	100.0	632	100.0	100.0	100.0
**Age at first sexual intercourse (1)**								
≤16	731	75.2***	46.2**	40.7	210	57.2	35.2	40.1
17–18	450	24.0	27.7	29.2	160	41.3	33.3	29.2
19+	410	0.7	26.1	30.1	144	1.5	31.6	30.7
Total	1592	100.0	100.0	100.0	514	100.0	100.0	100.0
**Parity**								
None	768	90.8	57.0	12.1	290	93.8	74.1	19.5
1–2 children	557	8.9	39.1	33.1	168	6.2	23.6	34.8
3 or more	607	0.2	3.9	54.8**	173	0.0	2.3	45.6
Total	1932	100.0	100.0	100.0	632	100.0	100.0	100.0
**Visited by CHW**								
No	1828	94.8	92.3	95.4	623	98.6	99.3	98.3
Yes	104	5.2*	7.7**	4.6**	9	1.4	0.7	1.7
Total	1932	100.0	100.0	100.0	632	100.0	100.0	100.0

** p<.05 ** p < .01 *** p < .001, p-value comparing slum and non-slum by age group.*
